# Cytomegalovirus-Mediated T Cell Receptor Repertoire Perturbation Is Present in Early Life

**DOI:** 10.3389/fimmu.2020.01587

**Published:** 2020-09-30

**Authors:** Meriem Attaf, Julia Roider, Amna Malik, Cristina Rius Rafael, Garry Dolton, Andrew J. Prendergast, Alasdair Leslie, Thumbi Ndung'u, Henrik N. Kløverpris, Andrew K. Sewell, Philip J. Goulder

**Affiliations:** ^1^Division of Infection and Immunity, Cardiff University School of Medicine, Cardiff, United Kingdom; ^2^Systems Immunity Research Institute, Cardiff University, Cardiff, United Kingdom; ^3^Human Immunodeficiency Virus Pathogenesis Programme, Doris Duke Medical Research Institute, Nelson R. Mandela School of Medicine, University of KwaZulu-Natal, Durban, South Africa; ^4^Africa Health Research Institute, Nelson R. Mandela School of Medicine, University of KwaZulu-Natal, Durban, South Africa; ^5^German Centre for Infection Research, Munich, Germany; ^6^Department of Infectious Diseases, Ludwig-Maximilians-University, Munich, Germany; ^7^Department of Paediatrics, University of Oxford, Oxford, United Kingdom; ^8^Zvitambo Institute for Maternal and Child Health Research, Harare, Zimbabwe; ^9^Centre for Genomics and Child Health, Blizard Institute, Queen Mary University of London, London, United Kingdom; ^10^Department of International Health, Johns Hopkins Bloomberg School of Public Health, Baltimore, MD, United States; ^11^Infection and Immunity, University College London, London, United Kingdom; ^12^The Ragon Institute of Massachusetts General Hospital, Massachusetts Institute of Technology and Harvard University, Boston, MA, United States; ^13^Virology and Immunology, Max Planck Institute for Infection Biology, Berlin, Germany

**Keywords:** cytomegalovirus, T cell receptor, T cell receptor repertoire, superdominance, paediatric repertoire, repertoire dynamics, memory inflation, HLA-B^*^44:03

## Abstract

Human cytomegalovirus (CMV) is a highly prevalent herpesvirus, particularly in sub-Saharan Africa, where it is endemic from infancy. The T cell response against CMV is important in keeping the virus in check, with CD8 T cells playing a major role in the control of CMV viraemia. Human leukocyte antigen (HLA) B^*^44:03-positive individuals raise a robust response against the NEGVKAAW (NW8) epitope, derived from the immediate-early-2 (IE-2) protein. We previously showed that the T cell receptor (TCR) repertoire raised against the NW8-HLA-B^*^44:03 complex was oligoclonal and characterised by superdominant clones, which were shared amongst unrelated individuals (i.e., “public”). Here, we address the question of how stable the CMV-specific TCR repertoire is over the course of infection, and whether substantial differences are evident in TCR repertoires in children, compared with adults. We present a longitudinal study of four HIV/CMV co-infected mother-child pairs, who in each case express HLA-B^*^44:03 and make responses to the NW8 epitope, and analyse their TCR repertoire over a period spanning more than 10 years. Using high-throughput sequencing, the paediatric CMV-specific repertoire was found to be highly diverse. In addition, paediatric repertoires were remarkably similar to adults, with public TCR responses being shared amongst children and adults alike. The CMV-specific repertoire in both adults and children displayed strong fluctuations in TCR clonality and repertoire architecture over time. Previously characterised superdominant clonotypes were readily identifiable in the children at high frequency, suggesting that the distortion of the CMV-specific repertoire is incurred as a direct result of CMV infection rather than a product of age-related “memory inflation.” Early distortion of the TCR repertoire was particularly apparent in the case of the TCR-β chain, where oligoclonality was low in children and positively correlated with age, a feature we did not observe for TCR-α. This discrepancy between TCR-α and -β chain repertoire may reflect differential contribution to NW8 recognition. Altogether, the results of the present study provide insight into the formation of the TCR repertoire in early life and pave the way to better understanding of CD8 T cell responses to CMV at the molecular level.

## Introduction

Human cytomegalovirus (CMV) is a herpesvirus which is highly prevalent globally and endemic in sub-Saharan populations (1, 2). In most immunocompetent hosts, CMV is asymptomatic, and MHC class-I restricted CD8 T cell responses keep CMV viraemia and disease in check (3, 4). In the immunocompromised, however, CMV reactivation can have serious adverse effects, as seen in transplant patients under immunosuppressive regimens (5), in the context of AIDS (6, 7) and in the elderly (8, 9). During the 1980's, human immunodeficiency virus (HIV) pandemic, CMV co-infection was a leading cause of morbidity and mortality in HIV-infected individuals (10, 11). Presently, CMV remains a burden to human health. This is particularly true for vulnerable groups such as children with congenital CMV infection (12), and “frail” patients suffering from autoimmune disease—even if they are otherwise immunocompetent (12, 13).

The induction of protective immune responses throughout an individual's lifespan relies on the maintenance of a functional T cell pool, especially in the case of antiviral immunity where T cells play a critical role (14, 15). Indeed, T cell responses are instrumental in protecting against CMV and several studies suggest that much of the T cell receptor (TCR) repertoire may be dedicated to controlling CMV infection (16–18). The TCR repertoire is generated by the thymus early in T cell ontogeny [reviewed in (19)]. However, the thymus involutes from puberty and as a consequence, thymic output is gradually reduced. Despite thymic involution and the associated reduction in thymic emigrants which naturally occur with age, both the size and the composition of the T cell pool remain remarkably constant throughout human life, except in very old age (20, 21). The decline in thymic activity in the elderly is believed to affect the TCR repertoire in several ways. First, reduced thymic output depletes the T cell pool of newly-generated naïve T cells, leading to an accumulation of memory T cells, many of which will display an activated or exhausted phenotype [reviewed in (22)]. Second, memory T cells expand as a result of antigen exposure and homeostatic expansion, leading to a distortion of repertoire architecture, erosion of TCR diversity and oligoclonality (21, 23, 24). Lastly, this increasing inequality in clone sizes is thought to have functional consequences, which effectively translate to increased susceptibility to infectious disease (14). We recently described a paragon of virally-induced TCR repertoire distortion by showing that CMV-infected HLA-B^*^44:03 individuals possessed large oligo/monoclonal expansions of superdominant clones (25, 26). Thus, in this setting, the immunological space available to tackle pathogens other than CMV may be significantly compromised by the extent of such TCR repertoire skewing (27).

In contrast to old age, the architecture, stability and composition of the TCR repertoire in infancy remains poorly understood due to the scarcity of deep sequencing studies conducted with paediatric samples (28, 29). Several small-scale studies have looked at the composition of the foetal repertoire (30), although very few were carried out at the deep sequencing level (20). Moreover, how the repertoire is regulated in childhood years is remarkably understudied. Thus, how the absolute number, the clonal size and dynamics of TCRs are controlled between birth and puberty are largely unknown. CMV infection has been shown to negatively impact on the development of naïve CD4 and CD8 T cells in adolescents, indicating that major events occurring during childhood regulate the composition of the T cell pool (31). Whether and how the effect of CMV infection on the T cell pool is manifest at the level of the TCR repertoire remains unknown.

We previously showed that HLA-B^*^44:03 subjects mounted a robust CD8 T cell response against the immediate-early-2 (IE-2)-derived epitope NEGVKAAW (NW8) (25), with drastic clonal expansion of superdominant TCR clonotypes (26). This finding led us to the hypothesis that TCR superdominance may be a feature of “memory inflation” occurring as a result of gradual expansion of antigen-specific T cells over time. Memory inflation is characterised by lack of contraction after the acute phase of infection, often followed by late, gradual expansion of memory T cells. This phenomenon has been documented in murine CMV infection (32), as well as humans (33, 34). In this study we sought to characterise the TCR repertoires of HLA-B^*^44:03 HIV and CMV co-infected children and their mothers recruited as part of a longitudinal study spanning more than 12 years. Here, we extend our previous study to demonstrate that CMV-infected children display CMV-associated TCR repertoire distortion prior to their second birthday indicating that it was not the result of a gradual rise of memory T cell expansions akin to inflation. We also demonstrate for the first time that, much like the adult repertoire, the paediatric CMV-specific repertoire is remarkably dynamic and characterised by dramatic temporal fluctuations in TCR clonality. These results expand our current pool of knowledge on the regulation of the human TCR repertoire in early life.

## Materials and Methods

### Study Subjects

Peripheral blood mononuclear cells (PBMC) from four vertically HIV-1 C clade-infected children and their mothers were obtained between the years 2006 and 2017 from the Ithembalabantu Clinic and the Prince Mshiyeni Hospital in Durban, South Africa from the so-called PEHSS cohort (35, 36). In one family, PBMC were also obtained from a HIV-negative sibling. The mother-child pairs had been followed-up longitudinally for over 12 years at the time of manuscript preparation. All individuals were CMV-infected, HLA-B^*^44:03 individuals (Table 1). Written informed consent was obtained from all study participants. Additionally, assent to participate in the study was given directly by children in the appropriate age groups. Studies were approved by the Biomedical Research Ethics Committee, University of KwaZulu-Natal, Durban; and Research Ethics Committee, University of Oxford.

**Table 1:** Patient characteristics.

**PID**	**Relation**	**HIV status**	**DOB**	**HLA-A1**	**HLA-A2**	**HLA-B1**	**HLA-B2**	**HLA-C1**	**HLA-C2**	**Sampling date**	**Age**	**Phenotyping**	**Clonotyping**
35C	Child	Positive	23-Nov-04	02:05	30:04:00	42:01:00	44:03:00	02:02	17:01	15-Aug-06	1.7	Yes	Yes
										18-Jun-07	2.6	Yes	Yes
										19-Jan-10	5.2	Yes	Yes
										19-Aug-13	8.7	Yes	Yes
35M	Mother	Positive	14-May-83	29:02:00	30:04:00	15:10	44:03:00	02:02	03:04	25-May-06	23	Yes	Yes
										24-Jul-07	24.2	Yes	Yes
										18-Feb-08	24.8	Yes	Yes
										13-Oct-09	26.4	Yes	Yes
										19-Aug-13	30.3	Yes	Yes
76C	Child	Positive	01-Mar-05	29:02:00	68:02:00	14:01	44:03:00	07:01	08:02	18-Sep-06	1.5	No	Yes
										22-Jul-08	3.4	Yes	Yes
										20-Jul-10	5.4	Yes	Yes
										17-Jul-12	7.4	No	Yes
										19-Aug-14	9.5	Yes	Yes
										03-Jun-16	11.3	Yes	Yes
										20-Feb-17	12	Yes	Yes
76M	Mother	Positive	01-Nov-78	24:02:00	29:02:00	07:02	44:03:00	07:01	07:02	12-Dec-05	27.1	No	Yes
										27-Jun-06	27.7	Yes	Yes
										16-Jul-07	28.8	No	Yes
										30-Jan-08	29.2	Yes	Yes
										27-Aug-13	34.8	Yes	Yes
										19-Aug-14	35.8	Yes	Yes
										20-Aug-15	36.8	No	Yes
										03-Jun-16	37.6	Yes	Yes
										15-May-17	38.5	Yes	Yes
64C	Child	Positive	23-Sep-04	29:02:00	30:02:00	41:02:00	44:03:00	07:01	17:01	03-Nov-05	1.1	Yes	Yes
										31-May-06	1.7	Yes	Yes
										08-Jun-10	5.7	Yes	Yes
										28-Jul-14	9.8	Yes	Yes
										30-Oct-15	11.1	Yes	Yes
64M	Mother	Positive	22-Aug-87	03:02	29:02:00	35:01:00	44:03:00	04:01	07:01	05-Apr-05	17.6	Yes	Yes
										18-Apr-07	19.7	Yes	Yes
										09-Sep-08	21.1	Yes	Yes
										30-Nov-10	23.3	Yes	Yes
										28-Jan-14	26.4	Yes	Yes
										28-Jan-16	28.4	Yes	Yes
64S	Sibling of 64C	Negative	05-May-06	ND	ND	ND	44:03:00	ND	ND	30-Oct-15	6.7	Yes	Yes
21C	Child	Positive	06-Sep-05	02:05	33:01:00	42:01:00	44:03:00	02:10	17:01	19-Apr-06	0.6	No	No
										23-Oct-13	8.1	No	No
										08-Oct-15	10.1	No	No
										11-Jan-16	10.3	No	No
										21-Jul-17	11.9	Yes	Yes
21M	Mother	Positive	09-Jul-73	02:05	33:01:00	42:01:00	44:03:00	02:10	17:01	5-Feb-03	29.6	No	No
										26-Jul-05	32.1	No	No
										12-Dec-05	32.4	No	No
										2-Jun-06	32.9	No	No
										4-Jul-06	33.0	No	No
										21-Jul-17	44.1	Yes	Yes

### Viral Load Measurement

Viral load measurement was performed using the COBRA AmpliPrep COBAS TaqMan HIV-1 Test version 2.0 (Roche, South Africa) (range = 20 copies/mL to 10 million copies/mL) and the NucliSens Version 2.0 Easy Q/Easy Mag (Biomérieux, South Africa) assay (range = 20 copies/mL to 10 million copies/mL). For measurement of CMV viral loads, DNA was extracted from 200 μL plasma using the Roche High Pure Viral Nucleic Acid Kit (Roche Diagnostics GMbH). CMV PCR and viral load measurement was undertaken using the LightCycler® CMV Quant Kit (Roche Diagnostics GMbH) on a LightCycler® 2.0 instrument (limit of detection 235 copies/mL; 95% confidence interval 153–500 copies/mL).

### Peptide—Major Histocompatibility Complex (pMHC) Multimer Staining and Cell Sorting

pMHC tetramers were generated as previously described (25). Cryopreserved PBMC from each patient were stained with PE-conjugated peptide-MHC tetramers for 30 min. After washing, surface antibodies and near-IR Live/Dead marker (Invitrogen, Paisley, UK) were added and after 20 min incubation a last washing step performed. The samples were sorted immediately after on BD FACSAria (BD Biosciences, Paisley, UK) and HLA-B^*^44:03/NW8 tetramer+ CD8+ cells (referred to as tet+ cells thereafter) were collected in 300 μL of RLT lysis buffer supplemented with 5-mercaptoethanol (QIAGEN, Hilden, Germany). Flow cytometry data was analysed using FlowJo version 9.9.5. All antibody clones used in this study are further described in Supplementary Table 1.

### TCR Sequencing

RNA extraction was carried out using the RNEasy Micro kit (Qiagen, Hilden, Germany) as previously described (26, 37). Briefly, cDNA was synthesised using the 5′/3′ SMARTer kit (Takara Bio, France) according to the manufacturer′s instructions. The SMARTer approach used a Murine Moloney Leukemia Virus (MMLV) reverse transcriptase, a 3′ oligo-dT primer and a 5′ oligonucleotide to generate cDNA templates flanked by a known, universal anchor sequence. A reverse primer specific for the TCR-α or the TCR-β constant region (CαR1 5′ CCATAGACCTCATGTCTAGCACAG-3′ or CβR1 5′-GAGACCCTCAGGCGGCTGCTC-3′, Eurofins Genomics, Germany) was then used together with an anchor-specific forward primer (Takara Bio, France) in the following PCR reaction: 2.5 μL template cDNA, 0.25 μL High Fidelity Phusion Taq polymerase, 10 μL 5X Phusion buffer, 0.5 μL DMSO (all from Thermo Fisher Scientific, UK), 1 μL dNTP (50 mM each, Life Technologies, UK), 1 μL of each primer (10 μM), and nuclease-free water for a final reaction volume of 50 μL. Subsequently, 2.5 μL of the first PCR products were used to set up a nested PCR as above, using a nested set of primers flanked with Illumina index sequences (CαR2 5′-GGTGAATAGGCAGACAGACTTGTC-3′ or CβR2 5′-TGTGTGGCCAGGCACACCAGTGTG-3′, immediately followed by the Illumina index sequence, Eurofins Genomics, Germany). For both PCR reactions, cycling conditions were as follows: 5 min at 94°C, 30 cycles of 30 s at 94°C, 30 s at 63°C, 90 s at 72°C, and a final 10 min extension at 72°C. The final PCR products were loaded on a 1% agarose gel and purified with the QIAEX II gel extraction kit (Qiagen, Germany). Purified products were pooled and sequenced on an Illumina MiSeq instrument using the MiSeq v2 reagent kit (Illumina, UK). The efficiency and accuracy of the present method was evaluated using an artificial repertoire consisting of a total of 25 T cell clones of known TCR-β chain sequence, spiked into PBMCs isolated from an HLA-A2+ buffy coat (Supplementary Figure 1). RNA was then extracted and TCRs sequenced as described above using an Illumina MiSeq platform. The artificial repertoire was divided into two biological replicates, each sequenced twice. The overlap between the biological replicates was assessed using Sorensen′s coefficient (Supplementary Figure 1A):

$$\begin{array}{l} Sor=\frac{2\;|n1\;\cap \;n2|}{\left|n1|+|n2\right|} \end{array}$$

Where |n1| is the number of clonotypes in the first biological replicate, and |n2| the number of clonotypes in the second biological replicate.

The clones were spiked at frequencies ranging from 1 to 0.0001%, and the concordance between the expected and the observed frequencies were evaluated (Supplementary Figure 1B). A rarefaction curve was also built based on the cumulative read count in order to assess the depth of sequencing (Supplementary Figure 1C).

### TCR Sequence Analysis

TCR sequence analysis was performed using MiXCR (38). MiXCR employs a built-in library of reference germline variable (V), diversity (D), junctional (J), and constant (C) gene loci from the ImMunoGeneTics (IMGT) database (imgt.org). The IMGT nomenclature for TCR gene segments was used throughout the study. All aligned, in-frame, antigen-specific TCR clonotypes are available in the VDJDB repository at vdjdb.cdr3.net (39). By convention, the TCR third complementarity-determining region (CDR3) is written from the cardinal Cys residue to the conserved, J-encoded Phe residue. Low quality clonotypes were filtered out by omitting sequences represented by <4 reads. Clonotypes representing <0.1% of all sequencing reads were considered non-specific clonotypes (stemming from non-specific binding to peptide-MHC tetramer) and were also omitted for analysis. Wheel charts, or “circos,” representing gene usage were generated with the web-based tool vdjviz (40). The total number of TCR sequences does not correlate with the number of sorted tet+ cells or with the number of filtered reads (Supplementary Figure 2).

### Statistical Analysis

#### Repertoire Entropy (Diversity)

TCR diversity is measured by the Shannon entropy index (H′):

$$\begin{array}{l} H^{\prime }=-\overset{n}{\underset{i=1}{\sum }}p_{i}\ln\left(p_{i}\right)\; \end{array}$$

Where p_i_ is the frequency of the i^th^ clonotype in a population of n clonotypes.

A high H′ is indicative of high diversity.

#### Repertoire Clonality (Evenness)

TCR repertoire clonality is given by the Shannon evenness index (J′):

$$\begin{array}{l} J^{\prime }=\frac{H^{\prime }}{ln\left(n\right)} \end{array}$$

Where H′ is Shannon entropy and *n* the number of clonotypes in the population.

J′ is undefined for monoclonal samples. Low J′ values approaching 0 indicate minimal evenness, as seen after clonal expansion of antigen-specific species. The maximal J′ value is 1, when all clonotypes have equal frequencies (i.e., the population is perfectly even).

#### Statistical and Graphical Analysis

All pairwise statistical tests were performed in Prism v7.0 (GraphPad, San Diego, USA). Data are presented as the mean ± standard deviation. Differences between mean values were assessed by Student's *t*-tests. For correlation analysis, Pearson's *r* was computed using Prism v7.0. *P*-values < 0.05 were considered to indicate a statistically significant difference.

## Results

### Children Raise High-Magnitude CD8 T Cell Responses Against NW8 in Early Life, Which Are Maintained Over Time

**Figure 1:**
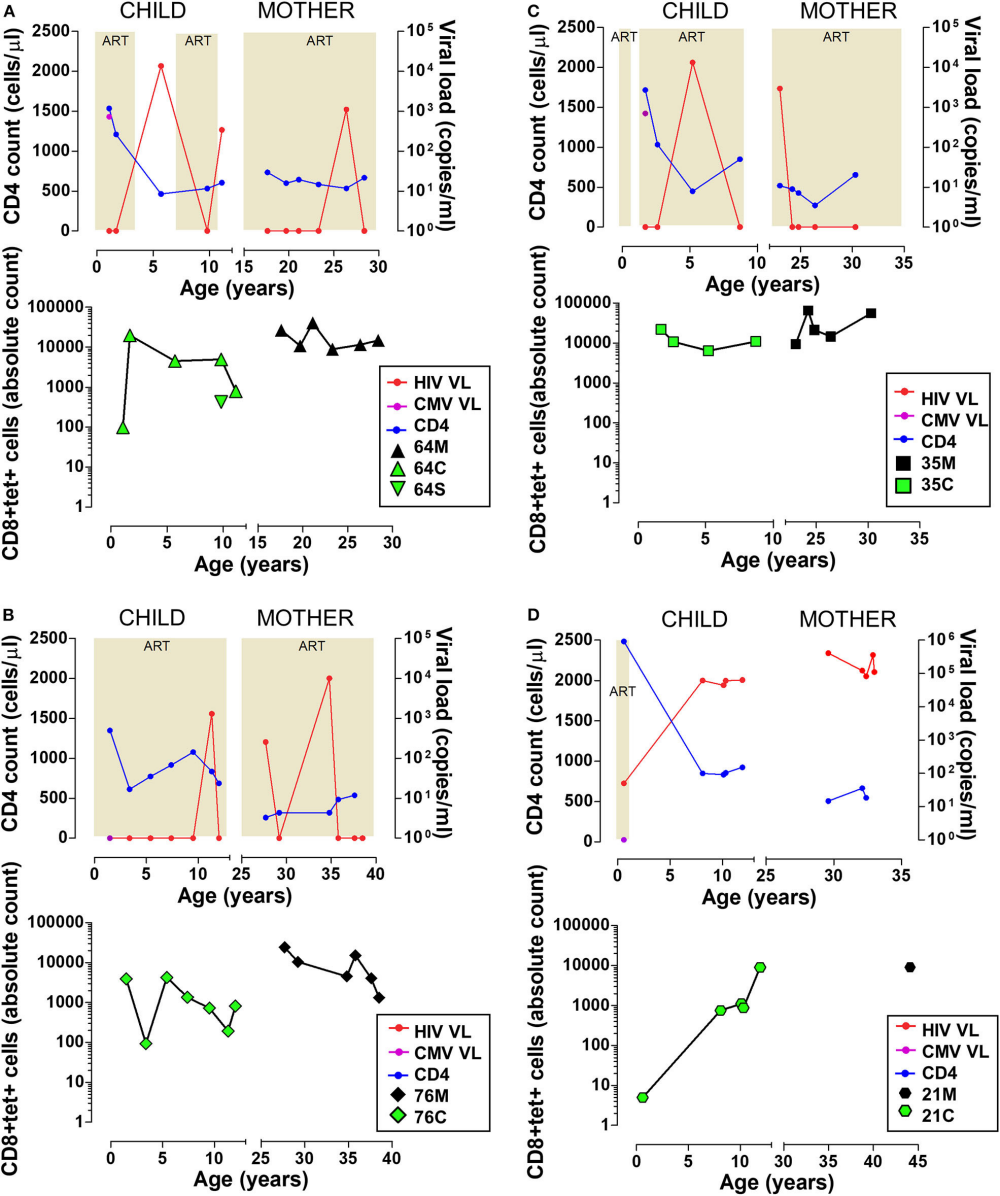
High magnitude HLA-B*44:03-restricted, NW8-specific T cell responses are detectable over 12+ years in four mother-child pairs. Clinical data and frequency of tet+ CD8 T cells are shown for four mother-child pairs. **(A)** 64C and 64M, as well as 64S, an HIV-uninfected sibling of 64C. **(B)** 76C and 76M. **(C)** 35C and 35M. **(D)** 21C and 21M. For 21M, only one timepoint was sampled and clinical data was not available. In all panels, data from the child is displayed on the left and data from the mother on the right side. Clinical data (top) includes the CD4 count in blue, HIV viral load in red, and CMV viral load in purple. The shaded areas indicate the periods when the subjects were on antiretroviral therapy. Longitudinal tetramer data (bottom) is shown as green symbols for children, and black symbols for mothers.

The immunodominant MHC class I-restricted CD8 T cell responses which control CMV are poorly characterised in sub-Saharan populations. We recently defined an immunodominant 8-mer peptide presented on HLA-B^*^44:03 which raised a very large immune response, amounting to almost 20% of CD8 T cells in one individual and a mean response of 3.4% of CD8 T cells across the cohort (25). We sought to characterise this HLA-B^*^44:03/NW8-specific CD8 T cell response in four HIV co-infected mother-child pairs in a longitudinal follow-up over 12 years (Figure 1). As TCR repertoire perturbation in CMV/HIV co-infected patients may partly depend on the duration and severity of immunodeficiency before ART, important indicators of HIV infection (CD4 count, HIV viral load and ART status) are illustrated in Figure 1, along with the absolute count of tet+ cells detected at each time point. Representative tetramer stains and phenotyping data are shown in Supplementary Figures 3, 4. The CMV viral load was determined for all four children at 3–4 months of age and was detectable in two of the four infants at this age, subject 64C and 35C, both of whom showed high-frequency NW8-specific CD8 T cell responses at that timepoint (3.6 and 6.3%, respectively), which remained high over the course of the 10 years follow-up. The other two children studied, 76C and 21C, were CMV-negative by viral load testing at 3–4 months (limit of detection, 235 CMV DNA copies/mL) and subsequent CMV-specific responses measured over the following decade were lower in magnitude. However, during the course of the study, every individual developed a strong NW8-specific CD8 T cell response, further underlining the high prevalence of CMV infection in sub-Saharan Africa as well as the immunodominance of the NW8 response in HLA-B^*^44:03 individuals. Thus, both children and adults raise and maintain high-magnitude CD8 T cell responses to the NW8 epitope over more than a decade of follow-up.

### TCR Bias in NW8-Specific Responses Is Manifest in Children and Adults Alike

**Figure 2:**
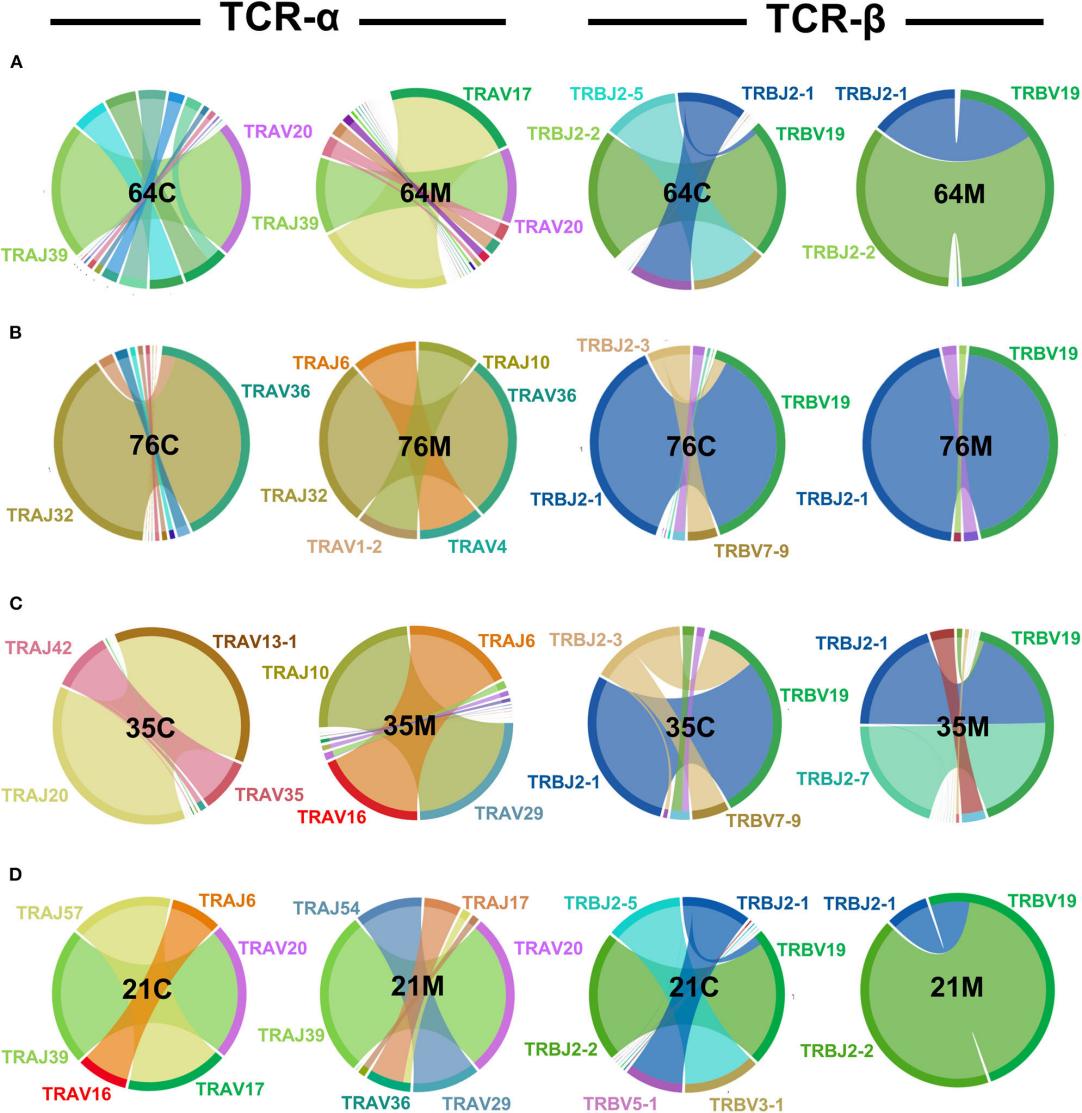
TCR-α and -β chain bias in NW8-specific CD8 T cell responses is manifest in children and adults alike. CD8 T cells were stained with HLA-B*44:03/NW8 tetramer and sorted for TCR-α and -β sequencing, as described in Materials and methods. TRAV-TRAJ (left) and TRBV-TRBJ (right) gene co-occurrence wheels are shown for CMV/HIV infected children, and their mothers. A single time point is shown for every individual, chosen so that the mothers and the children would be of similar age in their respective group. The size of the arcs is proportional to V or J frequency. The area joining any V-J pair is proportional to the co-occurrence frequency of that pair. Co-occurrence (“circos”) wheels were generated by VDJviz (40). Gene segments encoding dominant clonotypes are highlighted in colours matching the corresponding section in the wheel. Gene segment co-occurrence is shown for **(A)** the 64 family: 64M, the mother; 64C, the child. **(B)** the 76 family: 76M, the mother; 76C, the child. **(C)** the 35 family: 35M, the mother; 35C, the child. **(D)** The 21 family: 21M, the mother; 21C, the child.

TCR proteins are generated somatically by the discrete joining of V, D (for TCR-β chains) and J gene segments spliced to a constant region to form rearranged transcripts (41). Random joining of TCR gene segments would predict equal distribution of gene segments in a healthy repertoire at steady state. However, human infections typically give rise to narrow, predictable repertoires and unequal V and J gene usage (42–46). The NW8-specific CD8 T cell response is characterised by a consistent skew toward TRAV20 and TRBV19 usage in HLA-B^*^44:03 individuals, often paired with restricted J gene usage (26). To test whether this bias would be apparent in early life, we used peptide-MHC multimer staining and cell sorting of tet+ CD8 T cells and sequenced the TCR-α and TCR-β chain repertoire of the four mother-child pairs (25, 26, 37, 47, 48). We initially carried out this analysis at a single time point for every individual, chosen so that the children and the mothers would be similar in age, respectively (average of 1.9 and 26.4 years, respectively). Consistent with our previous report, the repertoire was heavily biased toward superdominant TRAV20 and TRBV19 clonotypes (Figure 2). In the case of the TCR-α chain, the germline TRAV20/TRAJ39 superclonotype made up a large proportion of the children (52% of all assigned reads) and the mother repertoires (nearly 100%) in two mother-child pairs (64C/M and 21C/M). Additional superdominant pairs were identified in the other subjects, such as TRAV36/TRAJ32, observed in mother-child pair 76M and 76C, or TRAV16/TRAJ6, which interestingly, was shared between unrelated individuals 35M and 21C. Also shared between unrelated individuals, was the TRAV17-TRAJ57 pair, seen in 64M and 21C. Similarly, for the TCR-β chain, TRBV19 clonotypes were highly prevalent. In all individuals TRBV19 was the most common V segment, paired with TRBJ2-1 or TRBJ2-2 and representing over 50% of all pairings across all individuals (and over 90% in three individuals). Of note, one HIV-negative individual, the sibling of 64C, also displayed a restricted TCR repertoire, further indicating that repertoire skewing is an inherent feature of CMV infection, rather than HIV-driven (Supplementary Figure 5). Thus, like the adults, the childhood repertoire is characterised by focused TRAV and TRBV gene usage in the NW8-specific CD8 T cell response.

While the superdominant clonotypes were largely shared across the subjects (children and adults alike), we also observed that certain subdominant V and J genes appeared in some cases specific to either the childhood or the adult repertoire, although the small number of subjects studied limits the conclusions that may be drawn from these preliminary data. This was the case for TRAV29, which was only seen in mothers (35M and 21M) and for TRAJ10 (35M and 76M). In the TCR-β chain, whereas the homologous TRBV19-TRBJ2.1 or TRBV19-TRBJ2.2 superclonotypes (one amino acid difference) were highly prevalent across the board, we found that certain TRBV and TRBJ genes, again, appeared restricted to children or to adults. TRBV3.1, for instance, was only detected in children (21C and 64C). This was also the case for TRBJ2.3 (21C and 64C) and TRBJ2.5 (35C ad 76C).

Thus, clonotypic superdominance from these data appears to be a universal feature of the NW8 response. By contrast, although study numbers here are small, there is a suggestion that the distribution of subdominant TCR chains may be a function of age and may differ between the paediatric and the adult repertoire.

### The Composition of the Childhood CMV-Specific Repertoire Fluctuates Over Time

**Figure 3:**
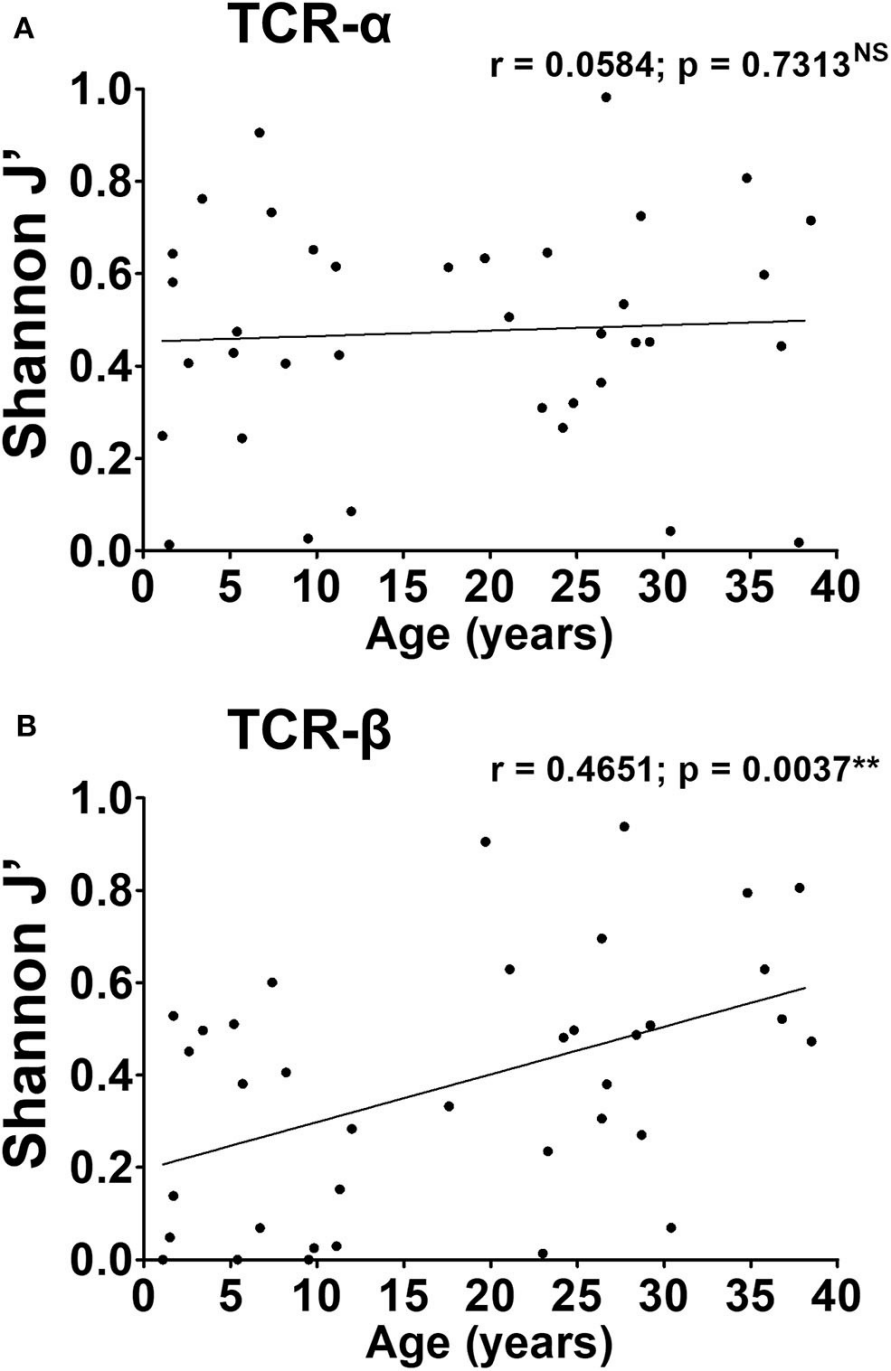
CMV-associated TCR repertoire perturbation arises in early life and is maintained over time. Oligoclonality of the TCR-α **(A)** and -β chain **(B)** repertoire was calculated as outlined in Materials and methods for each time point. J′ values of 0 have been used to represent monoclonal samples. Spearman's r was computed for every parameter and shown above the corresponding graph. *P*-values < 0.05 were considered significant (**p < 0.01, NS, not significant).

Having established that similar biases were apparent in paediatric and adult repertoires, we sought to characterise the TCR repertoires of three HIV/CMV co-infected mothers and their children for whom historical samples were available. To dissect the structure of the repertoire, we assessed the level of richness, diversity, and clonality of TCR-α and TCR-β chains (Figure 3).

The richness of the repertoire (i.e., the total number of TCR-α or TCR-β chain CDR3 clonotypes), varied widely across subjects but did not exceed 15 clonotypes for any individual (Supplementary Figures 6A,B). Children displayed limited richness compared to adults overall, although this was not statistically significant. No correlation was observed between the total number of TCR-α chains in the repertoire, and age (Pearson's *r* = 0.1228, not significant, Supplementary Figure 6A). Similarly, for TCR-β chains, although children appeared at first glance to display fewer clonotypes, there was no correlation between richness and age (Pearson's *r* = 0.2906; not significant, Supplementary Figure 6B). We also found that children, on average, displayed lower TCR-α Shannon entropy (mean H′= 0.62 ± 0.12) compared to the adults (mean H′ = 0.91 ± 0.08), indicative of lower diversity (*p* = 0.0402, Supplementary Figure 3C). This was also true for TCR-β (mean H′ = 0.48 ± 0.18 in children, vs. 0.91 ± 0.13 in adults, *p* = 0.0209, Supplementary Figure 3D). In fact, for TCR-β, there was a positive correlation between diversity and age (Pearson's *r* = 0.4278, *p* = 0.0083).

The level of clonality in TCR repertoires was assessed by Shannon evenness (J′). J′ is undefined for monoclonal samples. Low J′ values approaching 0 indicate minimal evenness, as seen after preferential clonal expansion of antigen-specific species. The maximal J′ value is 1, when all clonotypes have equal frequencies and thus the population is perfectly even. TCR clonality again varied widely with no distinct pattern emerging across all three pairs (Figure 3). For the TCR-α chain, the average J′ value was similar children and adults (mean J′ = 0.41 ± 0.06 in children and 0.51 ± 0.05 in adults, not significant). In the case of TCR-β, the difference in J′ between children and mothers was more pronounced (mean J′ = 0.26 ± 0.06 in children and 0.49 ± 0.06 in adults, *p* = 0.0091). This suggests a more uneven distribution of clonotypes, and more oligoclonal expansions in the childhood TCR-β repertoire, compared to the adult. Moreover, there was a positive correlation between J′ values and age, again reflecting a linear increase in repertoire evenness over time (Spearman's *r* = 0.4651; *p* = 0.0037).

We conclude that the architecture of the TCR-α repertoire is remarkably similar in children and adults. Indeed, the features that are typically associated with memory inflation, such as low diversity and skewing of the repertoire, are already present from a young age. In the childhood TCR-β repertoire, while some degree of oligoclonality and skewing exists, this tends to increase as a function of age. Altogether, these results argue against a gradual expansion of CMV specific populations spanning long periods of time. Rather, TCR superdominance in the CMV-specific repertoire is an acute phenomenon observable in young age soon after infection.

### TCR Skewing of NW8-Specific Responses Is Irreversible and Maintained Over 10+ Years

**Figure 4:**
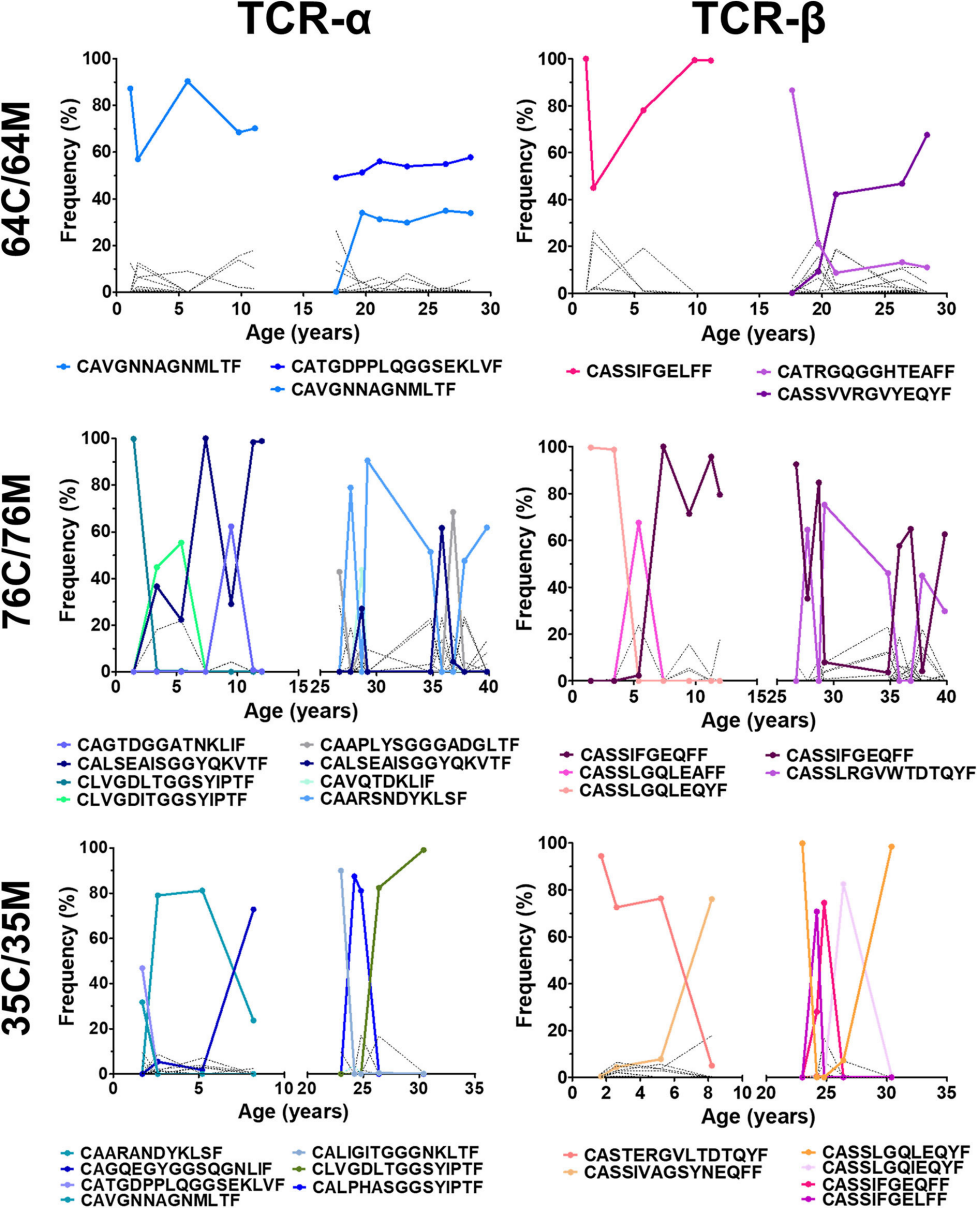
Clonotypic TCR superdominance is maintained in children and adults over time. The frequency of TCR-α (left) and TCR-β (right) is shown for three mother-child pairs monitored between 2005 and 2017. Superdominant clonotypes for each subject are shown in solid coloured lines. All other clonotypes are represented by solid black lines. A clonotype is considered superdominant if its frequency exceeds >30% at any point.

As mentioned above, all NW8-specific responses bear a set of superdominant clonotypes which are readily detectable both in children and adults. Having also demonstrated here that the childhood repertoire was surprisingly dynamic and fluctuated over time, we sought to establish how fluctuations in repertoire structure would affect the distribution of superdominant clonotypes (Figure 4). In some cases, we found that the frequency of superdominant clonotypes remained largely unchanged throughout the study period and this trend held true both in children and adults. These clonotypes were often the three most frequent and remained as such on multiple occasions (more than three timepoints). Strikingly, we found that these stable clonotypes were enriched with previously defined motifs, which were either germline (for TCR-α) or near-germline-encoded (for TCR-β) (26). One of the most stable superdominant clonotypes was the CAVGANNAGMLTF TCR-α chain (with the germline, J-encoded Asn/Ala/Gly or “NAG” motif), which represented between 60 and 90% of all assigned reads at every timepoint in subject 64C and over 30% at six consecutive timepoints in 64M. This clonotype was also present and stable in other subjects, although at lower frequencies. The clonotypes with the J-encoded Gly/Gly/Ser (“GGS”) motif were equally stable. Most subjects had at least one GGS-containing clonotype. In one individual, 64M, the CATGDPPLQGGSEKLF sequence accounted for over 50% of all assigned reads on six consecutive occasions. In the case of the TCR-β chain, the stability of the NAG and GGS clonotypes was mirrored by the high prevalence of the CASSIFGELFF/CASSIFGEQFF sequences, which dominated at multiple timepoints in all individuals. Like the NAG and GGS clonotypes, these IF clonotypes often reached extremely high frequencies (nearly 100% on three separate occasions in subjects 64C and 76C for instance).

Thus, whereas the NW8-specific repertoire as a whole can fluctuate over time, germline or near germline-encoded superdominant clonotypes are persistent. We conclude that TCR superdominance in CMV-specific responses emerges in early life and is irreversible.

## Discussion

The thymus involutes with age and with it, T cell production is reduced; yet the composition of the T cell repertoire remains remarkably constant. At the extremes of the human lifespan, T cell diversity is severely compromised, compared to adults. Reduced diversity, in turn, is largely believed to create “holes” in the TCR repertoire, which are linked to increased susceptibility to infection.

Ageing is strongly linked with this gradual reduction in TCR diversity. By contrast, the composition and functionality of the T cell repertoire in infants and children is poorly understood. In particular, the impact of viral infection on the paediatric TCR repertoire is not known. The earliest attempt to dissect the young repertoire used CDR3 spectratyping as a surrogate measurement method of diversity in umbilical cord blood, vs. adult peripheral blood, showing for the first time that all Vβ families were represented in the neonate repertoire. The only difference observed, compared to the adult repertoire, was a skewing in CDR3 length distribution (49). However, the spectratyping method is nowhere near comprehensive enough to provide a high-resolution view of the TCR repertoire. pMHC dextramer-based magnetic enrichment of antigen-specific CD8 T cells from umbilical cord blood places the precursor frequency of virus-specific (influenza A, Epstein Barr Virus, or CMV epitopes) TCR-β chain clonotypes in the order of 1–10 cells, per 100,000 CD8 T cells (30). Nonetheless, how individual, antigen-specific T cell clones are maintained in peripheral circulation throughout childhood is still unclear.

Britanova et al. were first to document a comprehensive, deep sequencing study following various age groups, including children and young people aged 6–25 years (20). In this study, key TCR-β chain repertoire characteristics, such as normalised diversity and functional overlap showed a strong association with age. Umbilical cord and young blood samples showed a substantial degree of publicity, whereas adult and old samples were more divergent. The clonal size of CMV-associated TCR-β clonotypes also increased with age. Nonetheless, one important caveat in this study was that TCR-β clonotypes were considered CMV-associated based on a literature survey and not through physical detection. The CMV status of the cohort was unknown, making it difficult to infer a role for CMV infection in shaping the repertoire over time.

Recently, the evolution of TCR-β diversity was evaluated in the CD8 T cell response to the immunodominant HSV-1 epitope derived from glycoprotein B (HSV gB_498−505_) SSIEFARL in neonatal, adult, and old C57BL/6 mice (50). The main limitation of using the murine model of T cell aging is the known discrepancies in the homeostasis of T cell numbers between humans and mice. Indeed, whereas mice can rely on thymic output for the replenishment of T cell numbers and TCR diversity throughout their lifespan, homeostatic proliferation is the main player in the maintenance of T cells in humans (51). Thus, a full picture of the changes that occur in the antigen-specific repertoire across the human lifespan has yet to emerge, and animal models where both lifespan and homeostasis are vastly different may be of little relevance in this respect.

We recently showed that HLA-B^*^44:03 adults raised a high-magnitude CD8 T cell response against the novel CMV IE2-derived epitope NW8 (25, 26). In this study we used the same approach in order to characterise the CD8 T cell repertoire responding to the NW8 epitope in three HLA-B^*^44:03+ children and their mothers over a period spanning 12+ years, with the aim of dissecting the changes that may occur in the paediatric and adult CMV-specific response over time. It is now well-established that T cells mount a robust response against ancient herpesviruses like CMV and EBV, which are highly focused and highly shared (41–45). We found that the response to NW8 was no exception, with adult HLA-B^*^44:03 individuals raising a biased TCR-α and TCR-β repertoire characterised by the presence of superdominant clonotypes. Here, using a unique mini-cohort of three mother-child pairs, followed longitudinally over 12+ years, we sought to determine whether a paediatric repertoire would display such superdominance and generally whether the characteristics of the childhood repertoire would be distinct from the adult.

The magnitude of CMV-specific CD8 T cell responses after natural infection is unusually high and can represent up to 10 to 20% of the peripheral blood CD8 T cell memory compartment in adults (26, 52). The size of this memory pool is often maintained or can slowly increase over a period spanning decades, a phenomenon termed “memory inflation” (32, 53). The frequency of the NW8-specific CD8 T cell response, as measured by tetramer staining, is also extremely high, a phenomenon we have previously likened to memory inflation. However, in the absence of longitudinal quantitation of NW8-specific CD8 T cells, it was not possible to establish whether this remarkably large response was the result of a gradual inflation process associated with age.

Our main finding is that the paediatric repertoire shares the same superdominant clonotypes we previously described in the adults, although the frequency of any particular clonotype, even the superdominant ones, largely varied from one point to the next, and this was true for both age groups. This large variation in the frequency of superdominant clonotypes may be indicative of competition for structural features on HLA-B^*^44:03, whereby TCR clonotypes compete for this pMHC niche, allowing homeostatic expansion of a clonotype only at the expense of another. Notably, TCR richness did not correlate with the number of sorted cells or read count, which largely excluded the possibility that the clonotypic fluctuations reported in the present study were the result of noise associated with variability in sample size.

Despite these inter-individual variations, the paediatric cohort studied here generally displayed the same features we previously observed in adults. Children, like their mothers, raised a high-magnitude CD8 T cell response to NW8. This response was focused, as indicated by biased V and J gene usage in the TCR-α and TCR-β repertoires. In addition, previously identified superclonotypes, that is, clonotypes found at a frequency >30% at any time point, were also present in children. For TCR-α, we found no correlation between repertoire richness, diversity, or evenness and age. In particular, Shannon's H' and J′ values were already high in the children group and certainly within the same range as the adults, indicating that distortions in the TCR-α repertoire arise early in life. In the case of TCR-β, we observed a positive correlation between Shannon's H′ and J′ with age, suggesting that TCR-β repertoire distortions are present in children but continue to rise at least until young adulthood. The presence of superdominant clonotypes at high frequency in children, in some cases as early as 1.6 years of age, argues against a process of gradual inflation, which typically arises incrementally over a period spanning years, if not decades. Rather, high-frequency generation of superclonotypes in young age may be explained by convergent recombination and efficient antigen-driven selection of virus-specific TCRs (45, 54), since these clonotypes are public (26). Nevertheless, there are many ways our results can be reconciled with the notion of memory inflation. One possibility is that the timing of CMV infection dictates the changes that occur in the repertoire as a result. In children, homeostasis of T cell numbers still relies predominantly on thymic output. Thus, CMV seropositivity may have an acute effect on the composition of the repertoire, whereby the presence of CMV antigens causes certain clonotypes to preferentially seed the peripheral T cell pool. At the other end of the age spectrum, when CMV is contracted in the elderly, a time where T cell numbers are mostly governed by slow, homeostatic expansion, memory inflation may become the primary mechanism that maintains and expands the CMV-specific T cell population. Moreover, our observation of increased TCR-β chain oligoclonality and diversity over time is in line with a report suggesting that while CMV may distort the repertoire in the elderly, the repertoire broadens to allow effective immune responses to some extent (55, 56). However, whether the repertoire distortions reported in the present study and in previous reports would reflect the composition of the CMV-specific repertoire in lymphoid tissue remains unclear. Indeed, a previous study demonstrated that repertoire overlap may not be as substantial in tissues, relative to what is typically observed in circulation (57).

Although we are aware of the limitations of this mini-cohort, these data provide insight into the dynamics of this immunodominant CMV-specific response in HLA-B^*^44:03 individuals. Monitoring the changes that occur over the human lifespan in the CMV-specific repertoire may not only provide insight into how TCR diversity is regulated throughout life but will also further our understanding of virus-specific responses in vulnerable groups, such as children with congenital CMV infection, or the elderly.

## Data Availability Statement

The datasets presented in this study can be found in online repositories. The names of the repository/repositories and accession number(s) can be found at: https://vdjdb.cdr3.net/, issue 313.

## Ethics Statement

The studies involving human participants were reviewed and approved by the Biomedical Research Ethics Committee of the University of KwaZulu-Natal (Durban) and the Research Ethics Committee of the University of Oxford (Oxford). Written informed consent to participate in this study was provided by the participants' legal guardian or next of kin.

## Author Contributions

The study was conceived by AS and PG and funded by grants to AS and PG. MA, JR, AM, CR, and GD did the experimental work that was supervised by AS, HK, TN, and PG. The data were analysed by MA, JR, AL, HK, AS, and PG. The manuscript was written by MA, JR, AL, HK, TN, AS, and PG. All authors contributed to the article and approved the submitted version.

## Conflict of Interest

The authors declare that the research was conducted in the absence of any commercial or financial relationships that could be construed as a potential conflict of interest.

## Supplementary Material

Supplementary Figure 1 TCR sequencing validation in a “spike-in” experiment. **(A)** Clonotypic overlap is represented as the Sorensen coefficient calculated between two biological replicates. This was carried for two sequencing runs; Run 1 is shown in white, Run 2 in grey. **(B)** The frequency of clones spiked at 0.0001, 0.001, 0.001, 0.01, 0.1, and 1% is shown. The expected frequency closely matches the observed frequency. **(C)** Rarefaction curve built from the total number of unique clonotypes detected as a function of cumulative reads. The rarefaction curve reaches saturation after 10 M reads, indicating that no new TCR clonotypes are detected with the inclusion of additional sequencing reads.

Supplementary Figure 2 Assessment of sample bias and noise in high-throughput sequencing of TCR-beta chains. The number of sorted tet+ cells does not correlate with TCR-beta **(A)** richness, **(B)** entropy, or **(C)** evenness. The number of processed reads does not correlate with TCR-beta **(D)** richness, **(E)** entropy, or **(C)** evenness.

Supplementary Figure 3 NW8 tetramer staining and CCR7/CD45RA differential expression. Representative flow cytometry plots are shown for every mother child pair: **(A)** 64C and 64M; **(B)** 76C and 76M; **(C)** 35C and 35M; **(D)** 21C and 21M. In each panel, HLA-B^*^44:03-NW8 tet+ CD8 T cells (gated on live CD3-positive T cells) are shown on the left. CCR7 vs. CD45RA expression is shown on the right.

Supplementary Figure 4 T cell activation and exhaustion in NW8-specific responses fluctuates over time. Longitudinal phenotypic T cell data are shown for **(A)** 64C and 64M, **(B)** 35C and 35M, **(C)** 76C and 76M. In each panel, HLA-DR expression (top row), PD-1 expression (upper middle row), and the frequency of T_EMRA_ cells (CD45RA^+^CCR7^−^; lower middle row) and T_EM_ cells (CD45RA^−^CCR7^−^; bottom row) are shown for tet+ and bulk CD8 T cells. Data from the children is displayed in dark green for tet+ and light green for bulk CD8 T cells (left side). Data from the mothers is in black for tet+, or grey for bulk CD8 T cells (right side).

Supplementary Figure 5 TCR-α and –β chain usage in a HIV-negative subject. TRAV-TRAJ (left) and TRBV-TRBJ (right) gene co-occurrence wheels are shown for patient 64S, a HIV-negative sibling of 64C. The size of the arcs is proportional to V or J frequency. The area joining any V-J pair is proportional to the co-occurrence frequency of that pair. Co-occurrence (“circos”) wheels were generated by VDJviz (40). Gene segments encoding dominant clonotypes are highlighted in colors matching the corresponding section in the wheel.

Supplementary Figure 6 TCR richness and diversity. Absolute repertoire richness is given by the total number of clonotypes and plotted as a function of age for the TCR-α **(A)** and the TCR-β chain **(B)**. TCR diversity was calculated as outlined in Materials and methods and displayed for TCR-α in **(C)** and TCR-β in **(D)**.

Supplementary Table 1 Antibodies used in the present study.
