# Corrigendum: Cytomegalovirus-Mediated T Cell Receptor Repertoire Perturbation Is Present in Early Life

**DOI:** 10.3389/fimmu.2020.633633

**Published:** 2020-12-21

**Authors:** Meriem Attaf, Julia Roider, Amna Malik, Cristina Rius Rafael, Garry Dolton, Andrew J. Prendergast, Alasdair Leslie, Thumbi Ndung’u, Henrik N. Kløverpris, Andrew K. Sewell, Philip J. Goulder

**Affiliations:** ^1^Division of Infection and Immunity, Cardiff University School of Medicine, Cardiff, United Kingdom; ^2^Systems Immunity Research Institute, Cardiff University, Cardiff, United Kingdom; ^3^Human Immunodeficiency Virus Pathogenesis Programme, Doris Duke Medical Research Institute, Nelson R. Mandela School of Medicine, University of KwaZulu-Natal, Durban, South Africa; ^4^Africa Health Research Institute, Nelson R. Mandela School of Medicine, University of KwaZulu-Natal, Durban, South Africa; ^5^German Centre for Infection Research, Munich, Germany; ^6^Department of Infectious Diseases, Ludwig-Maximilians-University, Munich, Germany; ^7^Department of Paediatrics, University of Oxford, Oxford, United Kingdom; ^8^Zvitambo Institute for Maternal and Child Health Research, Harare, Zimbabwe; ^9^Centre for Genomics and Child Health, Blizard Institute, Queen Mary University of London, London, United Kingdom; ^10^Department of International Health, Johns Hopkins Bloomberg School of Public Health, Baltimore, MD, United States; ^11^Infection and Immunity, University College London, London, United Kingdom; ^12^The Ragon Institute of Massachusetts General Hospital, Massachusetts Institute of Technology and Harvard University, Boston, MA, United States; ^13^Virology and Immunology, Max Planck Institute for Infection Biology, Berlin, Germany

**Keywords:** cytomegalovirus, T cell receptor, T cell receptor repertoire, superdominance, paediatric repertoire, repertoire dynamics, memory inflation, HLA-B∗44:03

## Incorrect Author Name

In the original article, an author name was incorrectly spelled as Andrew J. Predergast. The correct spelling is Andrew J. Prendergast.

The authors apologize for this error and state that this does not change the scientific conclusions of the article in any way. The original article has been updated.

