# Supplementary figures and images for: Cytomegalovirus-Mediated T Cell Receptor Repertoire Perturbation Is Present in Early Life
Source: Front Immunol. 2020 Sep 30;11:1587. doi: 10.3389/fimmu.2020.01587 (PMC7554308; doi:10.3389/fimmu.2020.01587)

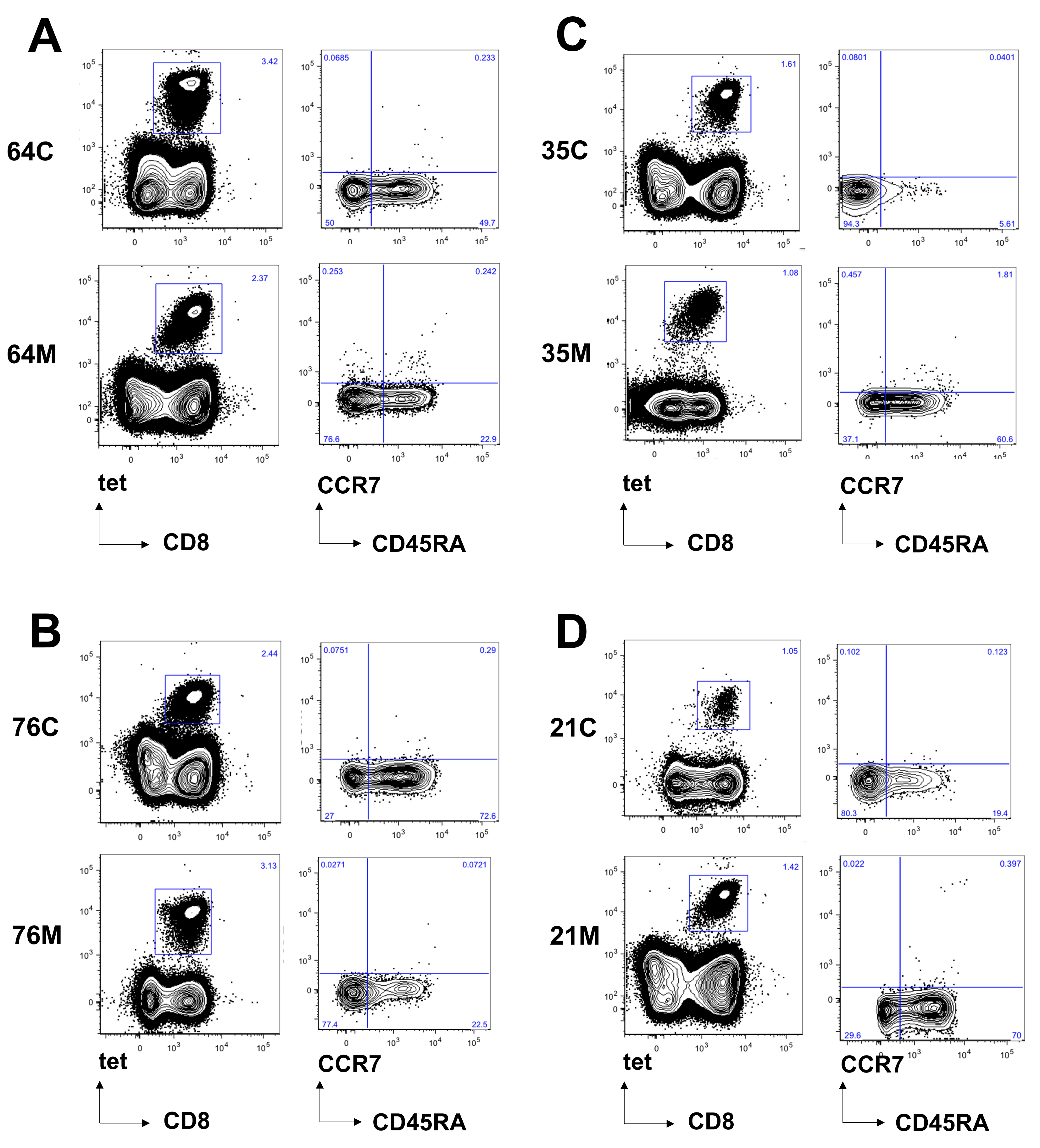

Supplement: Supplementary Figure 1 — TCR sequencing validation in a “spike-in” experiment. (A) Clonotypic overlap is represented as the Sorensen coefficient calculated between two biological replicates. This was carried for two sequencing runs; Run 1 is shown in white, Run 2 in grey. (B) The frequency of clones spiked at 0.0001, 0.001, 0.001, 0.01, 0.1, and 1% is shown. The expected frequency closely matches the observed frequency. (C) Rarefaction curve built from the total number of unique clonotypes detected as a function of cumulative reads. The rarefaction curve reaches saturation after 10 M reads, indicating that no new TCR clonotypes are detected with the inclusion of additional sequencing reads. [file Image_1.TIF]

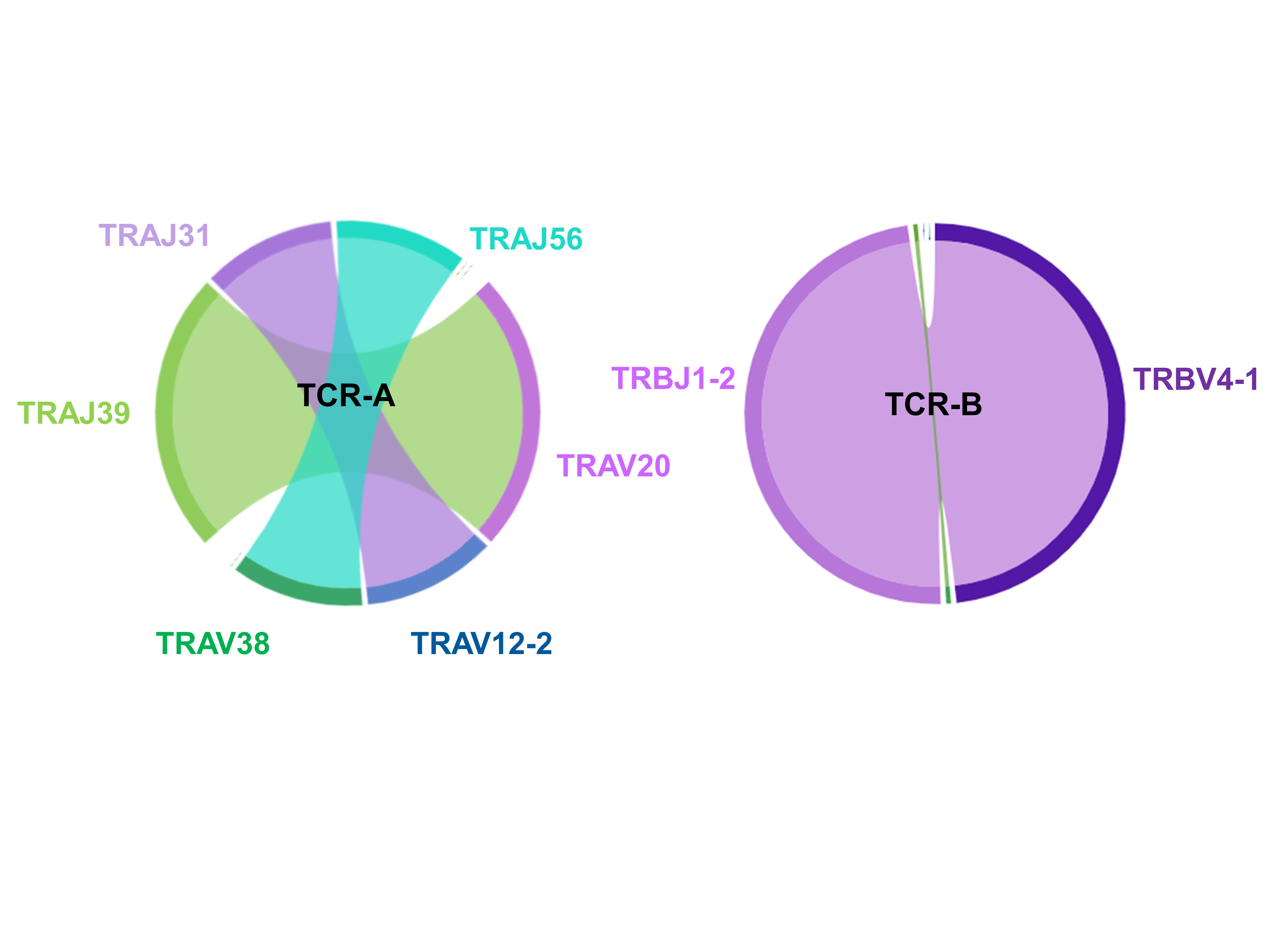

Supplement: Supplementary Figure 2 — Assessment of sample bias and noise in high-throughput sequencing of TCR-beta chains. The number of sorted tet+ cells does not correlate with TCR-beta (A) richness, (B) entropy, or (C) evenness. The number of processed reads does not correlate with TCR-beta (D) richness, (E) entropy, or (C) evenness. [file Image_2.TIF]

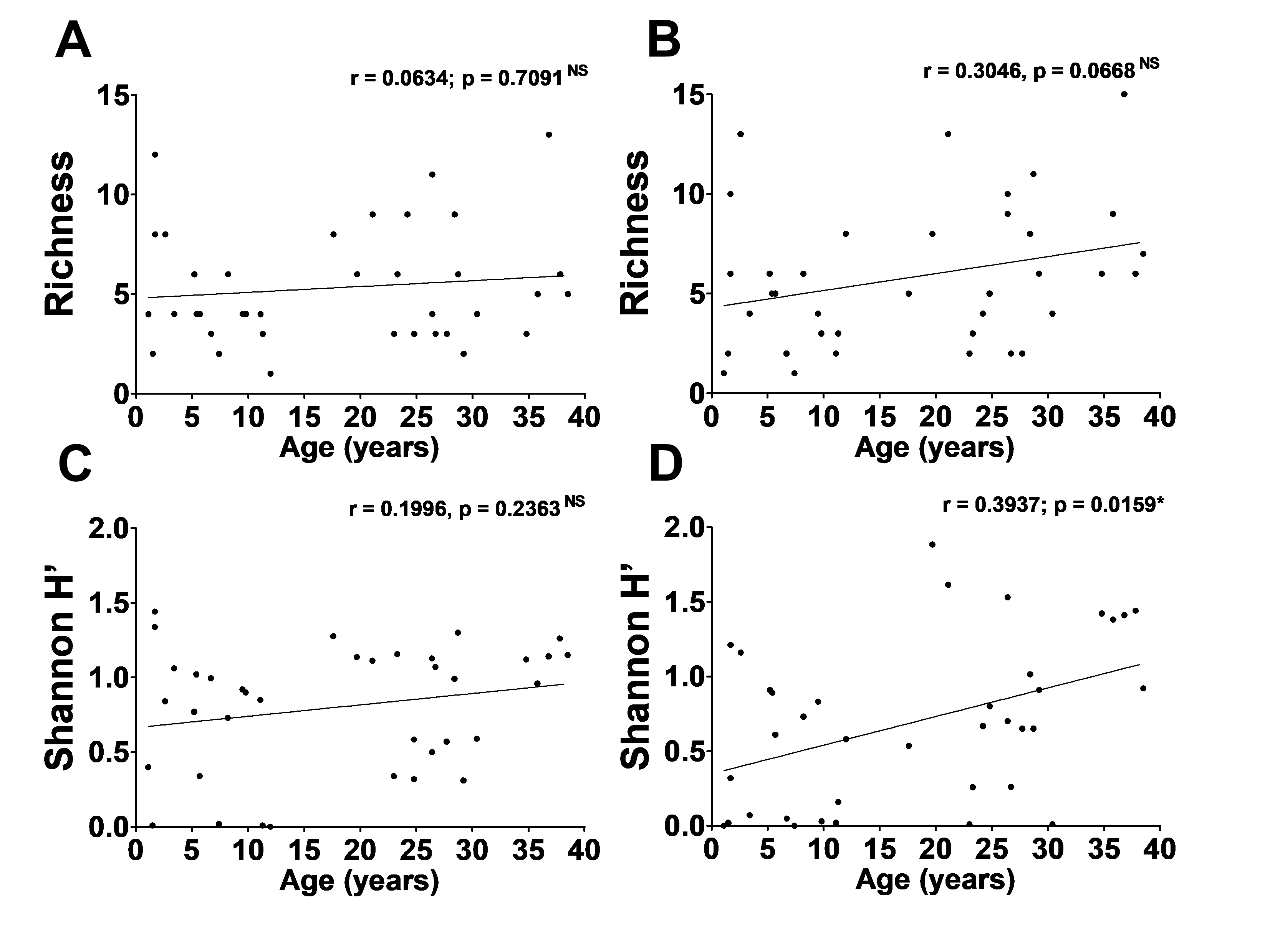

Supplement: Supplementary Figure 3 — NW8 tetramer staining and CCR7/CD45RA differential expression. Representative flow cytometry plots are shown for every mother child pair: (A) 64C and 64M; (B) 76C and 76M; (C) 35C and 35M; (D) 21C and 21M. In each panel, HLA-B*44:03-NW8 tet+ CD8 T cells (gated on live CD3-positive T cells) are shown on the left. CCR7 vs. CD45RA expression is shown on the right. [file Image_3.TIF]

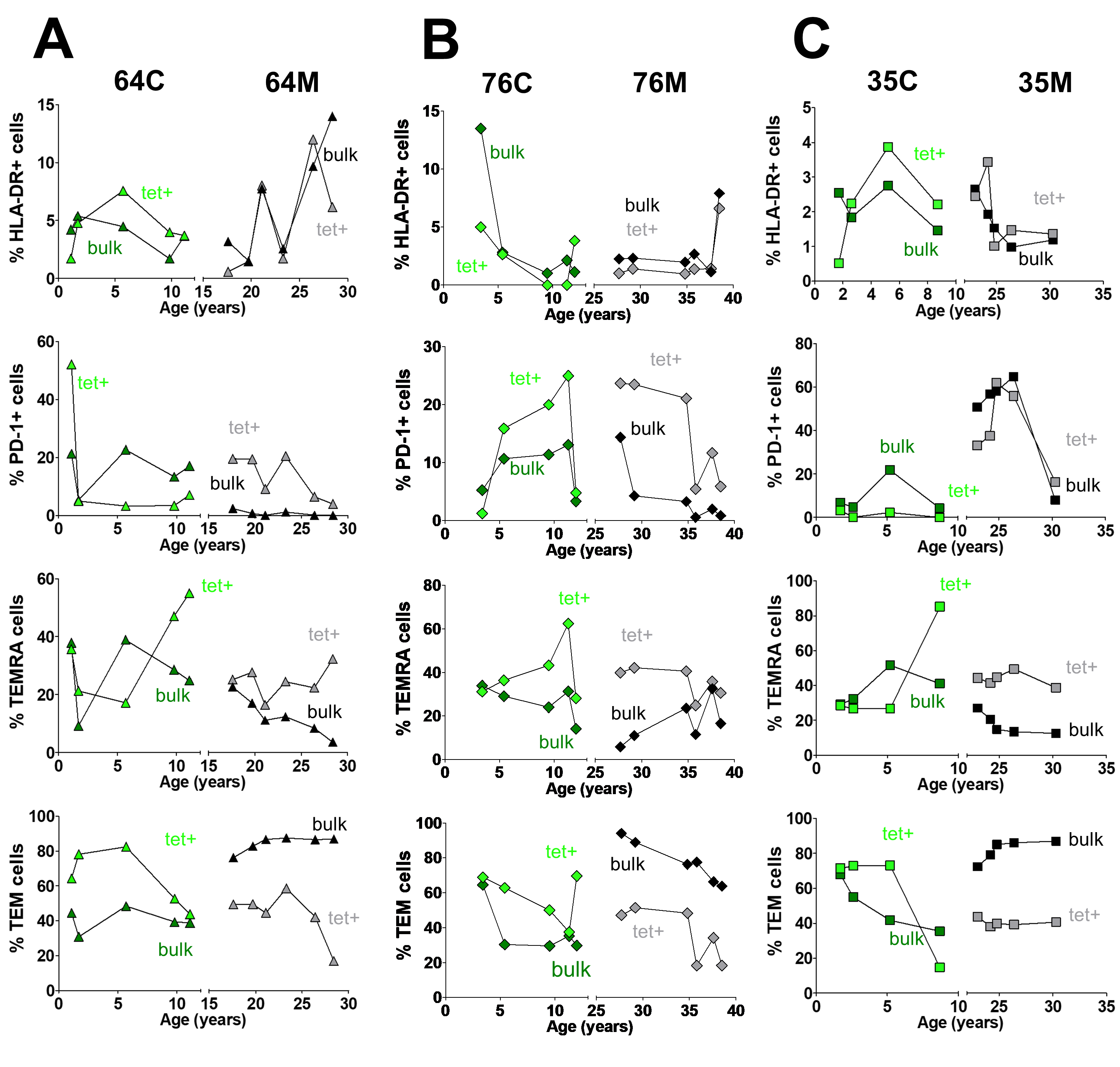

Supplement: Supplementary Figure 4 — T cell activation and exhaustion in NW8-specific responses fluctuates over time. Longitudinal phenotypic T cell data are shown for (A) 64C and 64M, (B) 35C and 35M, (C) 76C and 76M. In each panel, HLA-DR expression (top row), PD-1 expression (upper middle row), and the frequency of TEMRA cells (CD45RA+CCR7−; lower middle row) and TEM cells (CD45RA−CCR7−; bottom row) are shown for tet+ and bulk CD8 T cells. Data from the children is displayed in dark green for tet+ and light green for bulk CD8 T cells (left side). Data from the mothers is in black for tet+, or grey for bulk CD8 T cells (right side). [file Image_4.TIF]

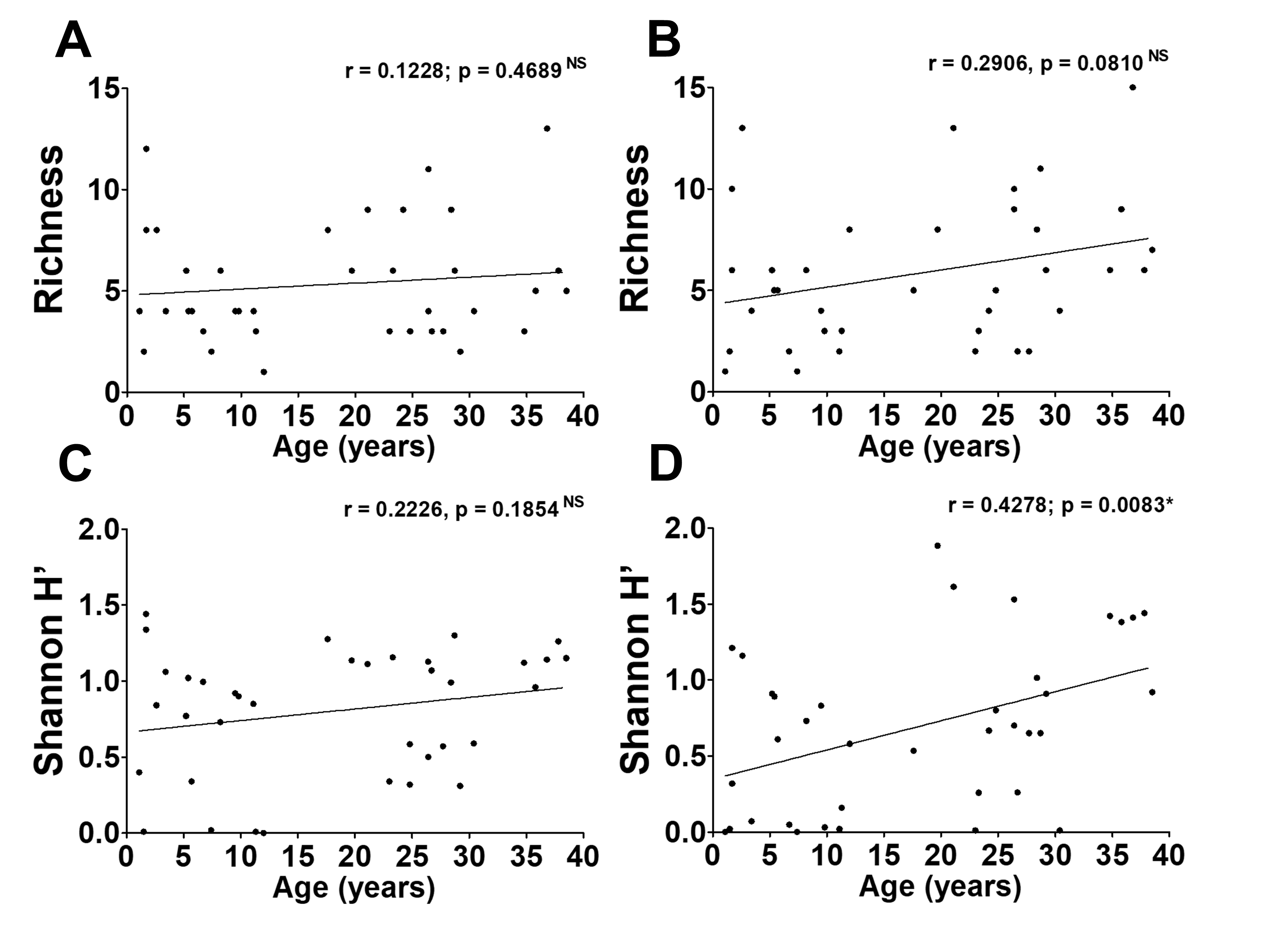

Supplement: Supplementary Figure 6 — TCR richness and diversity. Absolute repertoire richness is given by the total number of clonotypes and plotted as a function of age for the TCR-α (A) and the TCR-β chain (B). TCR diversity was calculated as outlined in Materials and methods and displayed for TCR-α in (C) and TCR-β in (D). [file Image_6.TIF]
