# Supplementary material for: Cytomegalovirus-Mediated T Cell Receptor Repertoire Perturbation Is Present in Early Life
Source: Front Immunol. 2020 Sep 30;11:1587. doi: 10.3389/fimmu.2020.01587 (PMC7554308; doi:10.3389/fimmu.2020.01587)
Supplement: Supplementary Table 1 — Antibodies used in the present study. [file Table_1.docx]

Supplementary Table 1.

| **Antigen** | **Fluorochrome** | **Clone** | **Supplier** |
| --- | --- | --- | --- |
| CD8 | BUV 395 | RPA-T8 | BD |
| CD4 | BUV 496 | SK3 | BD |
| PD1 | BV 421 | EH12.1 | BD |
| CD45 | V500 | Hi30 | BD |
| CD3 | BV 785 | OKT3 | Biolegend |
| HLA-DR | FITC | G46-6 | BD |
| CCR7 | PerCp-Cy5-5 | G043H7 | Biolegend |
| CD127 | PE-Cy7 | R34.34 | Beckmann Coulter |
| CD95 | PE-CF594 | DX2 | BD |
| CD45RA | AlexaFluor 700 | HI100 | Biolegend |
